# Transcriptome Analysis of the Initial Stage of Acute WSSV Infection Caused by Temperature Change

**DOI:** 10.1371/journal.pone.0090732

**Published:** 2014-03-04

**Authors:** Yumiao Sun, Fuhua Li, Zheng Sun, Xiaojun Zhang, Shihao Li, Chengsong Zhang, Jianhai Xiang

**Affiliations:** Institute of Oceanology, Chinese Academy of Sciences, Qingdao, China; Uppsala University, Sweden

## Abstract

White spot syndrome virus (WSSV) is the most devastating virosis threatening the shrimp culture industry worldwide. Variations of environmental factors in shrimp culture ponds usually lead to the outbreak of white spot syndrome (WSS). In order to know the molecular mechanisms of WSS outbreak induced by temperature variation and the biological changes of the host at the initial stage of WSSV acute infection, RNA-Seq technology was used to analyze the differentially expressed genes (DEGs) in shrimp with a certain amount of WSSV cultured at 18°C and shrimp whose culture temperature were raised to 25°C. To analyze whether the expression changes of the DEGs were due to temperature rising or WSSV proliferation, the expression of selected DEGs was analyzed by real-time PCR with another shrimp group, namely Group T, as control. Group T didn’t suffer WSSV infection but was subjected to temperature rising in parallel. At the initial stage of WSSV acute infection, DEGs related to energy production were up-regulated, whereas most DEGs related to cell cycle and positive regulation of cell death and were down-regulated. Triose phosphate isomerase, enolase and alcohol dehydrogenase involved in glycosis were up-regulated, while pyruvate dehydrogenase, citrate synthase and isocitrate dehydrogenase with NAD as the coenzyme involved in TCA pathway were down-regulated. Also genes involved in host DNA replication, including DNA primase, DNA topoisomerase and DNA polymerase showed down-regulated expression. Several interesting genes including crustin genes, acting binding or inhibiting protein genes, a disintegrin and metalloproteinase domain-containing protein 9 (ADAM9) gene and a GRP 78 gene were also analyzed. Understanding the interactions between hosts and WSSV at the initial stage of acute infection will not only help to get a deep insight into the pathogenesis of WSSV but also provide clues for therapies.

## Introduction

White spot syndrome (WSS) is the most devastating virosis threatening the shrimp culture industry worldwide [1]. The causative agent, white spot syndrome virus (WSSV), is an enveloped virus with a circular double strand DNA of about 300 kb, belonging to a new virus family *Nimaviridae* [2], [3]. The virus mainly infects animals of Decapoda and causes high lethality to shrimp [4]. Its outbreak depends on the interactions among the pathogen, host and environment. The interaction between WSSV and the hosts has been a research focus in recent years. Many host membrane proteins such as Rab7, β-integrin and chitin-binding protein showed interactions with WSSV envelope proteins *in vitro* and were proposed as possible WSSV receptors [5]–[7]. Shrimp STAT, Relish and Dorsal were annexed by WSSV to enhance viral immediate-early genes [8]–[11]. However, these studies are far from enough to illustrate the mechanisms of WSSV pathogenesis.

Environment has a profound impact on the outbreak of WSS. Water temperature is considered to be one of the most important environmental factors for shrimp. Field survey showed that in seasons with temperature lower than 20°C or higher than 30°C, the outbreak of WSS is abated; and temperatures between 22 to 30°C allow WSSV to replicate at a much higher rate [12], [13]. Studies in laboratory also verified the above phenomenon that temperature is crucial in determining WSSV proliferation [14]–[17]. Apoptosis might contribute to the increased survival of infected shrimp maintained at a hyperthermia state [18]. The main effect of hyperthermia on subcuticular epithelial cells was to reduce the expression of WSSV genes rather than to directly induce host genes that might contribute to control the infection [19]. A recent report showed that NAD-dependent aldehyde dehydrogenase (ALDH) and Hsp70 both play an important role in the inhibition of WSSV replication at high temperature [20]. Till present, there is no report about the initiation mechanisms of WSSV acute infection induced by temperature variation.

*Exopalamon carincauda* Holthuis has the potential to be an ideal experimental animal for crustacean with the virtues stated before [21]. In this study, shrimp *E. carincauda*, cultured at 18°C, were challenged with WSSV, followed by temperature rising to 25°C to mimic the state transition of the infected shrimp in culture ponds as demonstrated previously [12], [13]. It aims to get a better understanding of the biological changes of shrimp at the initial stage of the rapid propagation of WSSV and explore the initiation mechanisms of the acute infection caused by temperature rising.

## Materials and Methods

### 1 Maintenance of Experimental Shrimp and Viral Inocula Preparation

Experimental shrimp (*E. carincauda*) were hatched in our lab. They were detected to be WSSV free through one-step PCR as described previously [22]. These shrimp were maintained in natural sea water with continuous aeration and were fed with commercial food pellet twice a day.

WSSV was prepared and quantified as previously described [21]. The WSSV solution, serially diluted to 700 copies/µl with PBS, was used as inocula.

### 2 Challenge Experiment and Sampling

**Figure 1:**
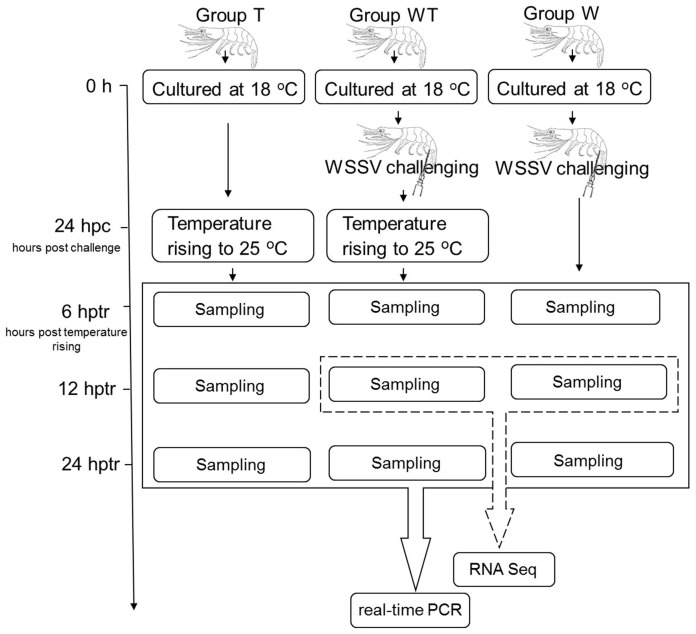
The experimental scheme. Three groups (Group T, Group W and Group WT) of shrimp were cultured at 18°C. Cephalothorax samples from 7 shrimp of the three groups were respectively collected as samples at 0 hour. For Group W, shrimp were challenged with WSSV inocula and kept at 18°C continuously. For Group WT, shrimp were also challenged with WSSV and the culture temperature was raised to 25°C at 24 h post challenge (hpc). For Group T, shrimp suffered no WSSV challenge. However the culture temperature of Group T was raised to 25°C in parallel with Group WT and then the two groups were maintained at 25°C thereafter. At 6, 12 and 24 hours post temperature rising (hptr), cephalothoraxes samples of 7 shrimp from each group were collected. Besides cephalothorax, pleopods were also collected from Group W and Group WT. Cephalothorax samples from Group W and Group WT at 12 hptr were subjected to Illumina sequencing.

Three groups, respectively named as Group T, Group W and Group WT, were set with 40 shrimp in each group. These shrimp, with an average length of 3.31±0.33 cm and an average weight of 0.51±0.17 g, were cultured at 18°C. Cephalothorax samples from 7 shrimp of the three groups were respectively collected as samples at 0 hour. For Group W, each shrimp was injected intramuscularly with WSSV inocula at a dose of 7000 copies, and kept at 18°C continuously. For Group WT, each shrimp was also injected with WSSV inocula at a dose of 7000 copies, and the culture temperature was raised to 25°C within 3 hours at 24 h post injection. They were kept at this temperature thereafter. For Group T, shrimp suffered no WSSV challenge. However the culture temperature of Group T was raised to 25°C within 3 hours at the same time with that of Group WT and then they were cultured at 25°C thereafter. At 6, 12 and 24 hours post temperature rising (hptr), 7 shrimp from each group were sacrificed and their cephalothoraxes were collected. Besides cephalothorax, pleopods were also collected from shrimp of Group W and Group WT. All the samples were immediately preserved in liquid nitrogen. The experimental scheme described above was shown in Fig. 1.

### 3 DNA Extraction and Viral Load Quantification

DNA was extracted from pleopod samples using plant genomic DNA kit (Tiangen, China) following the instructions. Protease K was used additionally at a final concentration of 2 µg/µl for sample digestion. Extracted DNA was subjected to electrophoresis in 1% agarose gel and quantified by NanoDrop 1000 spectrophotometer (Thermo Fisher Scientific Inc., USA).

Four-fold serially diluted solutions of a plasmid harboring the gene encoding the extra-cellular part of WSSV envelope protein VP28 were used as standard to generate a standard curve in the quantitative real-time PCR, performed with primers QVP28F and QVP28R (Table 1) to determine the viral loads in extracted DNA using a mastercycler ep realplex (Eppendorf, Germany), just as previously described [22].

**Table 1:** Primers used in this study.

Primer	Sequence (5′ to 3′)	Amplified sequences	Annealing temperature
18SF	TATACGCTAGTGGAGCTGGAA	18S	56°C
18SR	GGGGAGGTAGTGACGAAAAAT		
CL4068.C2F1	CCAAGAACGAAGAGACCATTTATAG	CL4068.Contig2_All	55°C
CL4068.C2R1	GCTGTCTCAGGTGCCCATAATAC		
U3409F1	GTTGGATGCCCCTCGTGTT	Unigene3409_All	55°C
U3409R1	GTTCTTCTTTCTGGATGACCGATAAT		
CL1901.C1F1	ACGAGTCGGAGACGGAGAT	CL1901.Contig1_All	53°C
CL1901.C1R1	GCAACCAGCCAACATAAGTAT		
U37574F1	CCTTGGCACTTTTTGTGACTGG	Unigene37574_All	54°C
U37574R1	CACTCCCTTTGGTGTTTATTCC		
U43802F1	ATCACTGGTCAGGAATATAATGAAAAG	Unigene42832_All	54°C
U43802R1	GCTGCCTATGAGTCGTTGCT		
CL2571.C1F1	GGATTGTGCGAACTTCCTGATGG	CL2571.Contig1_All	57°C
CL2571.C1R1	GCTGAGCCCTGAAACGGATTATGT		
U83F1	CACTCGCACAAACAAACAGG	Unigene83_All	54°C
U83F1	CTGCTTTAAGAAACAACCCGTAAG		
U20038F1	GGTATGCTGGTCTTTATTTCACTCG	Unigene20038_All	56°C
U20038R1	ACCTGGGTCTCTCTTCTTCATTTTAT		
CL2500.C1F1	CACGCAAAGAACAGCAACCTAC	CL2500.Contig1_All	56°C
CL2500.C1R1	GATACCCAAGCCGCCAGAG		
U7524F1	ATTCCCTAACTTGATGTCCCT	Unigene7524_All	54°C
U7524R1	GGTGATGCCATTCTTGATTC		
U42358F1	ATGTTGGCACAAACTTCCT	Unigene42358_All	50°C
U42358R1	TGGGCTTCAGCAAACCTAT		
U37466F2	CTCTTGGTAACTGCCATTCATT	Unigene37466_All	52°C
U37466R2	AGAATAGCGATTGAAGGAAGC		
U37705F1	CTGTCAGACTCCAGAACAATCC	Unigene37705_All	56°C
U37705R1	TCATAGCCAGAGCAGAGAGCATC		
U9162F1	AAAACTAATACAAAGAATGAGGTGCTGGAT	Unigene9162_All	57°C
U9162R1	TGTGATACGGTTGCCTTGGTCG		

### 4 RNA Extraction and cDNA Synthesis

Total RNA was extracted from cephalothorax samples with RNAisol reagent (Takara, Japan), assessed by electrophoresis in 1% agarose gel, quantified by NanoDrop 1000 spectrophotometer and treated with RNase free DNase I (Promega, USA) to remove the contaminating DNA. The cDNA synthesis were proceeded at 37°C for 2 hours with 4 µg RNA, 40 µM random primers (Sangon, China), 50 mM dNTP, 160 U RNasin (Promega, USA), 20 µl 5×M-MLV buffer and 800 µM-MLV (Promega, USA) in a total volume of 100 µl.

### 5 Illumina Sequencing

RNA samples of Group W and Group WT at 12 hptr were subjected to Illumina sequencing using the paired-end RNA-seq method (Bentley et al., 2008). Briefly, poly (A) mRNA was isolated from total RNA with Sera-mag oligo(dT) beads and fragmented to a length of 200 bp to 700 bp. cDNA was synthesized with these fragments and random hexamer primers. After end reparation, addition of poly (A) and ligation with sequencing adapters, fragments with suitable length were recovered from agarose gel and amplified by PCR for 15 cycles. The products were purified with MinElute column (Qiagen), followed by paired-end sequencing using Illumina HiSeq™ 2000.

### 6 Reads Assembly and Sequence Annotation

Transcriptome *de novo* assembly is carried out by program Trinity and TIGR Gene Indices clustering tools (TGICL) [23]. The obtained unigenes were annotated by BLASTx with the sequences available in NR database and Swiss-Prot databases (E-value<10^−5^).

### 7 Analyzing the Biological Changes of the Host from the Transcriptomic Data

The expression of unigenes was calculated as RPKM (Reads per Kilobase of exon model per Million mapped reads), followed by multiple hypothesis testing and FDR (False Discovery Rate) control. DEGs were screened with the two following standards. Firstly, the FDR should be no larger than 0.001. Secondly, the expression difference between two groups should be larger than 3 times or the expression difference had been testified by real-time PCR to be larger than 2 times.

GO (Gene Ontology, http://www.geneontology.org/) functional classification annotation for DEGs was carried out and the GO ontology “biological process” was analyzed. The up and down-regulated DEGs for GO analysis were listed in Files S1 and S2 separately.

Based on the annotation information, genes involved in the glycometabolism pathways including glycosis and tricarboxylic acid cycle pathway (TCA) as well as DNA replication were manually screened and compared between Group W and Group WT at 12 hptr.

### 8 Quantitative Real-time PCR Analysis of Selected Genes from the Transcriptome

Some interesting genes were manually screened. Quantitative real-time PCR was performed with primers in Table 1 to analyze the expression levels of the interesting genes in Group T, Group W and Group WT at 6, 12 and 24 hptr as well as to verify the transcriptomic data. The 18S rRNA gene, which served as a stably expressed reference, was quantified with primers 18SF and 18SR (Table 1).

### 9 Statistical Analysis

One-way ANOVA and *t* test were used to analyze the obtained data with SPSS 17.0 software (*P*<0.05 as the significant level).

## Results and Discussions

### 1 Mortality and the Viral Load Variations of Group W and Group WT

No mortality happened in Group T during the whole experiment course. In Group WT a few died shrimp began to emerge at about 24 hptr and then WSS broke out. No more death occurred in Group WT after 48 hptr with a final mortality of 94%. In Group W, no mortality appeared until 96 hptr, followed by the WSS outbreak beginning at 120 hptr. The situation in Group W stabilized at about 168 hptr and the final mortality was 96%. The data supported the previous reports that the temperature affected the WSSV infection and lower temperature could reduce WSSV pathogenicity [13], [15], [24], [25]. Lower temperature (18°C) could only delay the infection progression, rather than completely stopping the infection course.

**Figure 2:**
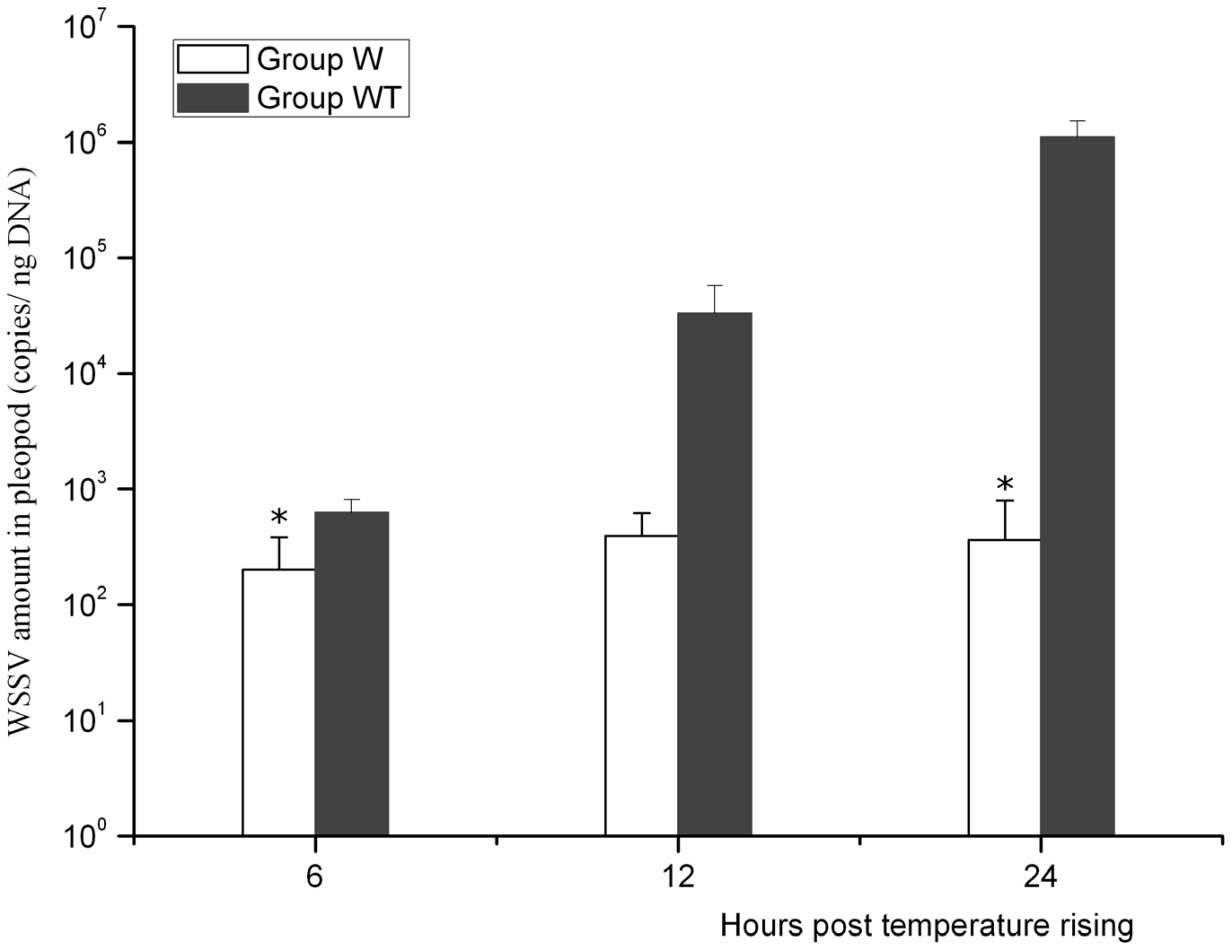
Viral loads in the pleopod of Group W and Group WT. DNA was extracted from pleopods of Group W and Group WT sampled at 6, 12 and 24 hours post temperature rising. And viral loads in extracted DNA were determined by quantitative real-time PCR with serially diluted solutions of a plasmid harboring the gene encoding the extra-cellular part of WSSV envelope protein VP28 as the standard sample to generate a standard curve. Significant differences between the two groups were indicated by asterisks (*P*<0.05).

WSSV loads in pleopod samples of Group W and Group WT were quantified by real-time PCR (Fig. 2). The WSSV amount in Group WT was 628.28±187.88 copies/ng DNA, 3.31×10^4^±2.45×10^4^ copies/ng DNA and 1.10×10^6^±4.28×10^5^ copies/ng DNA at 6, 12 and 24 hptr respectively, and *t* test analysis showed significant difference in the viral amount between samples at 24 hptr and that at 12 hptr (*P*<0.05). However it was only 201.86±182.08 copies/ng DNA, 395.06±226.99 copies/ng DNA and 364±434.82 copies/ng DNA in Group W at the corresponding time respectively and no significant difference was observed among the viral loads at the above time points by One-way ANOVA (*P*>0.05).

Previous study showed that the viral load in the pleopod at the light infection stage was at the level of no more than 10^3^ copies per ng DNA [22]. When the viral load in the pleopod ranged from 10^3^ to 10^5^ copies per ng DNA, the infection progressed in the logarithmic phase of WSSV replication, during which the WSSV load experienced a sharp leap from 4.16×10^3^ copies/ng DNA at 27 hours post infection (hpi) to 9.65×10^4^ copies/ng DNA at 30 hpi, suggesting a turning point from chronic infection to acute infection [22]. In the present study, Group W was indicated to be at the stage of latent infection when the viral loads in pleopods were not more than 10^3^ copies/ng DNA. The viral load in Group WT at 6 hptr showed no significant difference with that in Group W (*P*>0.05). However, the WSSV copy number in Group WT at 12 hptr was 3.31×10^4^±2.45×10^4^ copies/ng DNA, implying that Group WT was at the initial stage of the logarithmic phase of WSSV replication [22].

### 2 Reads Assembly and Sequence Annotation

Totally 54,156,824 clean reads in Group W and 54,258,768 clean reads in Group WT were obtained. After assembly, 61,886 unigenes were obtained (69,855 in Group W and 61,757 in Group WT) with an average length of 626 bp. The transcriptomic dataset was deposited in SRA database (No. SRR1105776) and the detailed information for sequence assembly was shown in Table 2.

**Table 2:** General information of the transcriptomes of shrimp from Group W and Group WT.

Dataset name	Group W	Group WT
Total raw reads (paired-end)	56,713,886	56,793,742
Total clean reads	54,156,824	54,258,768
Q20 percentage	98.68%	98.65%
N percentage	0.00%	0.00%
GC percentage	45.11%	45.05%
Contigs	141,836	131,106
Mean length (bp) of Contigs	270	268
N50 (bp) of Contigs	365	366
Unigenes	69,855	61,757
Mean length (bp) of Unigenes	494	501
N50 (bp) of Unigenes	675	698

Unigene annotation was performed by BLASTx to protein databases (E-value<10^−5^). A total of 25,399 unigenes (41.04% of all unigenes) were matched to known genes in NR database and 21,777 unigenes (35.19% of all unigenes) were matched to known genes in Swiss-Prot database. Other sequences have no homology to proteins in the above two databases. The phenomenon that in shrimp transcriptomic data, a large percentage of sequences with no matches in the protein database was also reported elsewhere and these unknown sequences, reflecting the limited knowledge that we have about the shrimp genomes [26], [27].

### 3 DEGs between Group WT and Group W

About 6,417 DEGs were obtained (File S3). 4687 DEGs were down-regulated in Group WT and the other 1,730 DEGs were up-regulated, of which 239 were WSSV genes (File S4), indicating the active transcription of WSSV genes at the initial stage of acute infection.

GO functional classification annotation for DEGs was carried out and 821 DEGs (643 down-regulated ones and 178 up-regulated ones in Group WT) had GO functional classification annotation. The DEGs were involved in various biological processes of the host, such as immune system process, metabolic process, energy production, organelle and cytoskeleton organization, cell cycle, signal transduction, death, cell adhesion, response to stimulus and so on, suggesting that the viral propagation after temperature rising had a comprehensive influence on the host.

#### 3.1 DEGs involved in the energy production and glycometabolic process

Go analysis showed that almost all the DEGs related to energy production were up-regulated in Group WT, suggesting an increased energy need and a boosted energy production (Table 3). This result was consistent with previous studies that proteins in electron transport and ATP synthesis such as cytochrome oxidase polypeptide, the alpha and beta subunits of mitochondrial ATP synthase, were up-regulated upon WSSV infection [28], [29]. The up-regulation of energy production could meet the energy requirements both of the host in the immune response to fight against viral infection and of the virus during the earlier phases of its rapid replication [29].

**Table 3:** GO analysis of the DEGs related to energy production.

GO terms	Percentage of down-regulated DEGs (%)	Percentage of up-regulated DEGs (%)
GO:0006119 oxidative phosphorylation	0.00	0.12
GO:0042773 ATP synthesis coupled electron transport	0.00	0.12
GO:0015980 energy derivation by oxidation of organic compounds	0.12	0.24
GO:0045333 cellular respiration	0.00	0.24
GO:0022900 electron transport chain	0.00	0.12
GO:0022904 respiratory electron transport chain	0.00	0.12

**Figure 3:**
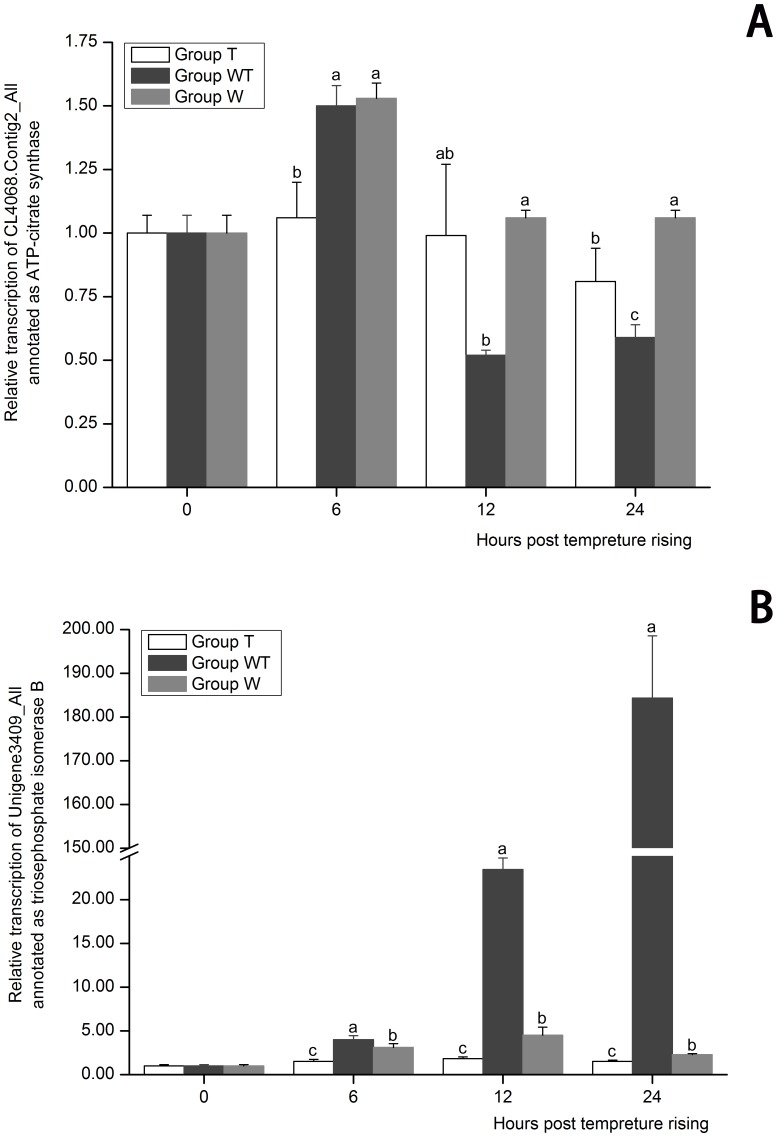
The expression profiles of CL4068.Contig2_All annotated as ATP-citrate synthase (A) and Unigene3409_All annotated as triosephosphate isomerase B (B). Their expression in Group T, Group W and Group WT at 6, 12 and 24 hours post temperature rising was tested by real-time PCR. The values are shown as mean±SE. Significant differences among the three groups are indicated by different characters (*P*<0.05).

Genes encoding the enzymes involved in the glycometabolism were manually screened to analyze the pathway transition of glycometabolism (File S5). Triose phosphate isomerase, enolase and alcohol dehydrogenase which were involved in glycosis, were all up-regulated in Group WT, suggesting that the glycosis was enhanced (Table 4). Pyruvate dehydrogenase, citrate synthase and isocitrate dehydrogenase with NAD as the coenzyme were down-regulated in Group WT, suggesting that a reduced glycometabolism level through the tricarboxylic acid cycle pathway (TCA) (Table 4). However the isocitrate dehydrogenase with NADP as the coenzyme was up-regulated in Group WT, which may be the compensatory expression for the inhibited expression of isocitrate dehydrogenase with NAD as the coenzyme. The expression profiles of ATP-citrate synthase (CL4068.Contig2_All) and triosephosphate isomerase B (Unigene3409_All) in the three groups were analyzed by real-time PCR to check whether the changes in glycometabolism were caused by temperature variation or WSSV proliferation (Fig. 3). The expression of ATP-citrate synthase showed no significant difference between Group T and Group W at 12 hptr (*P*>0.05), but its expression in Group WT at the same time was significantly lower than those in Group T and Group W. As for triosephosphate isomerase B, its expression in Group WT was remarkably higher than those in Group W and Group T (*P*<0.05) at both 12 hptr and 24 hptr. These data suggested that the TCA pathway was possibly weakened and the glycosis was enhanced due to the rapid replication of WSSV. It was reported that the normal flux in TCA cycle was disrupted by the infectious hypodermal and hematopoietic necrosis virus (IHHNV) in shrimp at least temporally and it was reckoned that the interruption of the Krebs cycle may favor the inducement of a higher lipogenic and cholesterogenic activity, which may ultimately be essential for the virus replication [30]. Metabolic changes resembling the Warburg effect in shrimp hemocytes were detected in the early stage of WSSV infection and it was proposed that the enhanced aerobic glycosis and ATP energy production favored the virus during the earlier phases of its replication cycle [31].

**Table 4:** Genes encoding enzymes involved in glycometabolism.

Gene ID	Gene annotation	E-value	Catalyzed pathway	Expression change
CL3945.Contig1_All	Pyruvate dehydrogenase protein X component, mitochondrial	9.00E-93	TCA	down
CL4068.Contig2_All	ATP-citrate synthase	0		
CL4965.Contig3_All	Isocitrate dehydrogenase [NAD] subunit gamma, mitochondrial	3.00E-153		
CL2802.Contig2_All	Isocitrate dehydrogenase [NADP], mitochondrial	0		up
Unigene3409_All	triosephosphate isomerase B	2.00E-137	glycosis	up
CL1501.Contig2_All	mitochondrial enolase superfamily member 1	0.00E+00		
Unigene18979_All	alcohol dehydrogenase class-3	2.00E-15		
Unigene28838_All	alcohol dehydrogenase class-3	7.00E-116		

#### 3.2 DEGs involved in the cell death process

**Figure 4:**
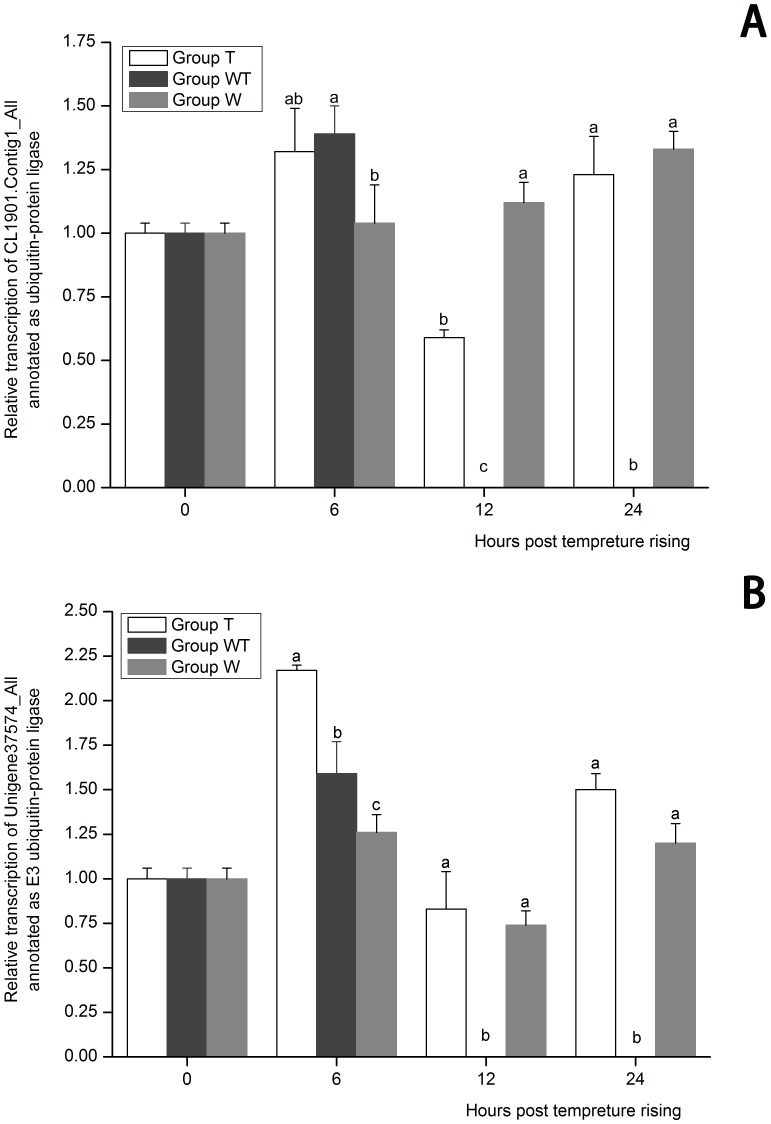
The expression profiles of CL1901.Contig1_All (A) and Unigene37574_All (B) both annotated as E3 ubiquitin-protein ligase. Their expression in Group T, Group W and Group WT at 6, 12 and 24 hours post temperature rising was tested by real-time PCR. The values are shown as mean±SE. Significant differences among the three groups are indicated by different characters (*P*<0.05).

GO analysis showed that DEGs related to positive regulation of cell death were down-regulated and DEGs related to anti-apoptosis were up-regulated in Group WT, implying that the virus inhibited the host cell death to benefit its own propagation (Table 5). Apoptosis has been regarded as a nonspecific defense mechanism against virus infection in eukaryotic cells, aborting virus multiplication by a premature lysis of infected cells [32]–[34]. Viruses have evolved various strategies to escape apoptosis of the host through viral anti-apoptosis genes to guarantee the production of enough viral progeny [35], [36]. Apoptotic-related proteins showed a high expression level in the virus resistant shrimp, suggesting that apoptosis might play important roles in shrimp immunity against WSSV infection [37], [38]. The enhancement of apoptotic activity effectively inhibited the WSSV infection in shrimp, resulting in decreased shrimp mortality, while apoptosis inhibitor increased the mortality of shrimp infected by WSSV [39]. WSSV showed the ability to modulate apoptosis of shrimp cell. Two WSSV anti-apoptosis proteins AAP-1 and WSSV222 were identified [40]–[43]. AAP-1 is a direct caspase inhibitor and WSSV222 functions through ubiquitin-mediated degradation and blocks apoptosis induced by the tumor suppressor-like protein [36]. In the present study, AAP-1 (Unigene30118_All) and WSSV222 (Unigene30290_All) were all remarkably up-regulated in Group WT at 12 hptr suggesting that they might be responsible for the inhibited apoptosis at the initial stage of WSSV acute replication. Most DEGs with annotation to E3 ubiquitin-protein ligase (9/10, File S6) were down-regulated. Among them, two E3 ubiquitin-protein ligase genes (CL1901.Contig1_All and Unigene37574_All) were further confirmed by real-time PCR (Fig. 4). Ubiquitin (Ub) E3 ligases were regarded as key regulators of cell death, which controlled many pro- and anti-apoptotic molecules at the protein level by Ub-dependent degradation or non-degradative ubiquitylation events [44]. The present data showed that the malfunction to control cell death happened at the initial stage of acute infection.

**Table 5:** GO analysis of the DEGs related to positive regulation of cell death and anti-apoptosis.

GO terms	Percentage of down-regulated DEGs (%)	Percentage of up-regulated DEGs (%)
GO:0010942 positive regulation of cell death	0.49	0.00
GO:0012502 induction of programmed cell death	0.24	0.00
GO:0043068 positive regulation of programmed cell death	0.37	0.00
GO:0043065 positive regulation of apoptosis	0.37	0.00
GO:0043066 negative regulation of apoptosis	0.49	0.24
GO:0070267 oncosis	0.12	0.00
GO:0048102 autophagic cell death	0.12	0.00
GO:0006916 anti-apoptosis	0.24	0.12

#### 3.3 DEGs involved in the cell cycle process

**Figure 5:**
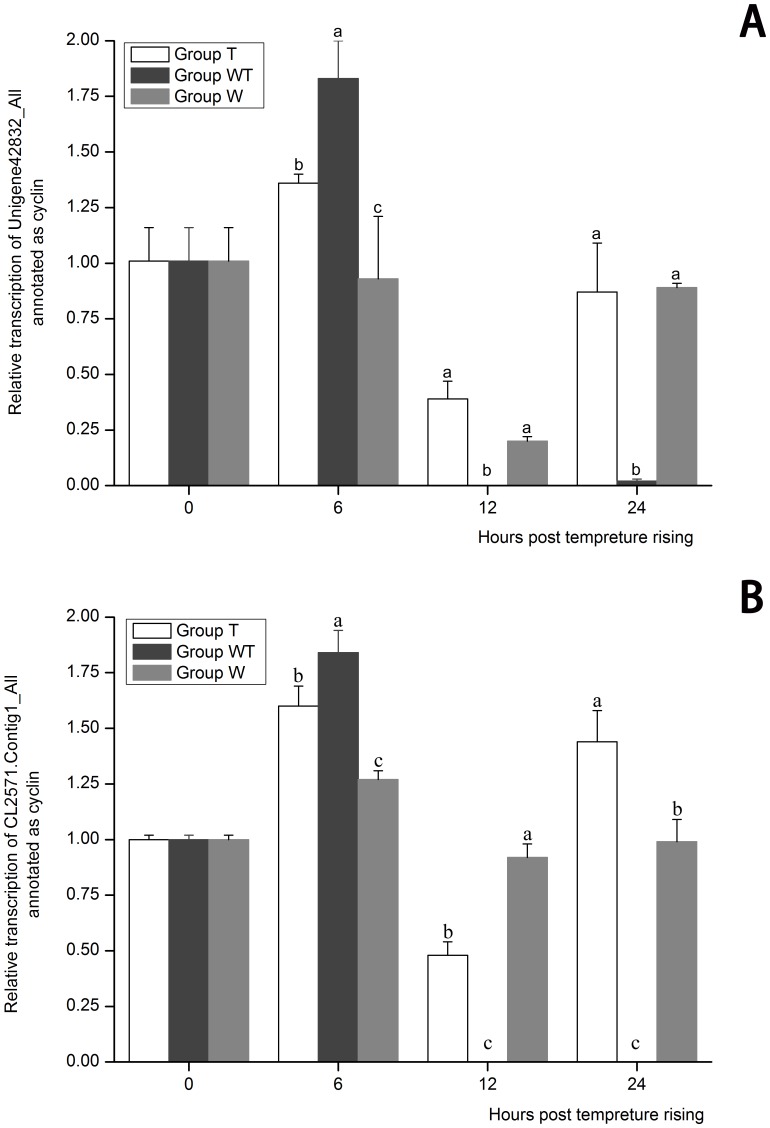
The expression profiles of Unigene42832_All (A) and CL2571.Contig1_All (B) both annotated as cyclin. Their expression in Group T, Group W and Group WT at 6, 12 and 24 hours post temperature rising was tested by real-time PCR. The values are shown as mean±SE. Significant differences among the three groups are indicated by different characters (*P*<0.05).

Go analysis displayed that almost all the DEGs related to cell cycle were down-regulated in Group WT, suggesting that the host cell proliferation was apparently inhibited (Table 6). The cell cycle consists of four phases, gap 1 (G1), synthesis (S), gap 2 (G2) and mitosis (M) in succession. In order to carry through this vital process orderly, a series of mechanisms have been developed and cyclins are a family of controller proteins. In the present study, 10 DEGs were annotated as cyclins, which were all down-regulated (File S7). The expression profiles of two cyclin genes (Unigene42832_All and CL2571.Contig1_All) in the three groups were further confirmed by real-time PCR (Fig. 5). The expression of both genes in Group WT at 12 hpi was nearly completely repressed, compared with that in Group T and Group W. These data further indicated that the inhibition of host cell proliferation was caused by WSSV; and the arrested state started at the initial stage of WSSV acute infection and lasted until 24 hptr at least when shrimp death began to occur. Some viruses were reported to be able to arrest host cells at certain cell phases with different mechanisms [45]–[49]. *Autographa californica* Nucleopolyhedrovirus (AcMNPV) arrested host cell at G2/M phase due to a virus-coded protein with cdc2-associated kinase activity [46]. Vpr protein of human immunodeficiency virus type 1 (HIV-1) activated cell cycle inhibitor p21/Waf1/Cip1, causing cellular arrest in G2/M phase [47]. Human cytomegalovirus (HCMV) induced a state of late G1 arrest and mouse hepatitis virus (MHV) infection resulted in an arrest at the G0/G1 phase [45], [48]. It was reported that alterations in cell cycle transit during virus infection might be very important for the virulence difference between Malignant fibroma virus (MV) and Shope fibroma virus (SFV) [50]. This may be also one of the causes of the high virulence of WSSV. The molecular mechanisms underlying the alteration in cell cycle transit needs to be further clarified to find a target for controlling the WSSV disease.

**Table 6:** GO analysis of the DEG related to cell cycle.

GO terms	Percentage of down-regulatedDEGs (%)	Percentage of up-regulatedDEGs (%)
GO:0007049 cell cycle	4.87	0.73
GO:0051726 regulation of cell cycle	1.83	0.00
GO:0045787 positive regulation of cell cycle	0.24	0.00
GO:0022402 cell cycle process	3.17	0.49
GO:0010564 regulation of cell cycle process	0.24	0.00
GO:0000278 mitotic cell cycle	2.80	0.49
GO:0007346 regulation of mitotic cell cycle	0.85	0.00
GO:0051329 interphase of mitotic cell cycle	1.10	0.24
GO:0000082 G1/S transition of mitotic cell cycle	0.37	0.24
GO:0031659 positive regulation of cyclin-dependent proteinkinase activity during G1/S	0.12	0.00
GO:0000084 S phase of mitotic cell cycle	0.49	0.00
GO:0007090 regulation of S phase of mitotic cell cycle	0.12	0.00
GO:0000085 G2 phase of mitotic cell cycle	0.12	0.00
GO:0000086 G2/M transition of mitotic cell cycle	0.12	0.00
GO:0010389 regulation of G2/M transition of mitotic cell cycle	0.12	0.00
GO:0000087 M phase of mitotic cell cycle	0.12	0.00
GO:0000132 establishment of mitotic spindle orientation	0.12	0.00
GO:0000216 M/G1 transition of mitotic cell cycle	0.49	0.00
GO:0000075 cell cycle checkpoint	0.73	0.00
GO:0007093 mitotic cell cycle checkpoint	0.49	0.00
GO:0051321 meiotic cell cycle	0.61	0.00

#### 3.4 DEGs involved in cellular DNA replication

**Figure 6:**
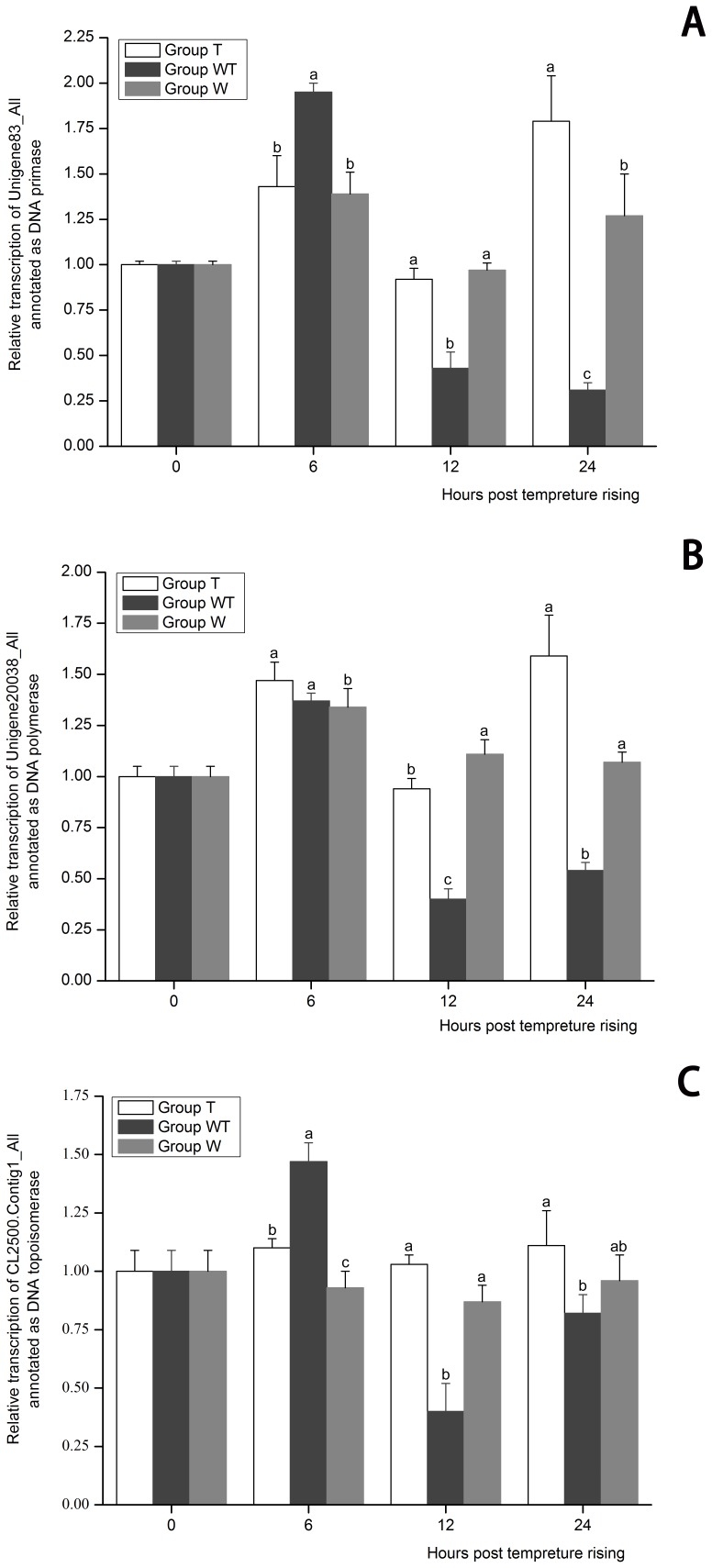
The expression profiles of Unigene83_All annotated as DNA primase (A), Unigene20038_All annotated as DNA polymerase (B) and CL2500.Contig1_All annotated as DNA topoisomerase (C). Their expression in Group T, Group W and Group WT at 6, 12 and 24 hours post temperature rising was tested by real-time PCR. The values are shown as mean±SE. Significant differences among the three groups are indicated by different characters (*P*<0.05).

Proteins participated in DNA replication were manually screened. DNA primase, DNA topoisomerase and DNA polymerase showed down-regulated expression in Group WT (see in the appended File S8), whereas the WSSV ORFs wsv514 and wsv447, which are homologous to eukaryotic DNA polymerase and helicase respectively, were remarkably up-regulated (Table 7). The expression profiles of Unigene83_All (a DNA primase), Unigene20038_All (a DNA polymerase) and CL2500.Contig1_All (a DNA topoisomerase) in the three groups at 6, 12 and 24 hptr were further detected by real-time PCR (Fig. 6). The expression of these three genes in Group WT at 12 hptr was significantly lower than that in Group T and Group W at 12 hptr (*P*<0.05), indicating that their expression had been repressed due to the rapid replication of WSSV. And as for Unigene83_All and Unigene20038_All, the repressed state lasted till 24 hptr at least when shrimp death began to occur. All these data indicated that WSSV proliferation inhibited the cellular DNA replication process and WSSV may rely on its own polymerase and helicase for DNA replication. The adverse effect on the cellular DNA synthesis in the infection course of other viruses such as frog virus 3, herpes simplex virus, HCMV and AcMNPV were also reported and the repressed state of cellular DNA replication ensures that the virus will have unrivalled access to precursors of DNA synthesis [45], [51]–[53]. The decline of cellular DNA synthesis during the infection of frog virus 3 and herpes simplex virus might be caused by the infecting particles themselves and does not require the synthesis of new RNA or protein [51], [52]. The mechanisms of the repressed cellular DNA synthesis during WSSV infection still need further study.

**Table 7:** Genes involved in DNA replication.

Gene ID	Gene annotation	E-value	Expression change in Group WT at 12 hptrcompared to Group W
Unigene83_All	DNA primase-like protein	4.00E-139	down
CL2500.Contig1_All	DNA topoisomerase 1-like	0	down
Unigene16622_All	putative prokaryotic DNA topoisomerase	1.00E-96	down
Unigene1672_All	DNA polymerase alpha catalytic subunit	2.00E-31	down
Unigene20038_All	DNA polymerase delta catalytic subunit	4.00E-59	down
Unigene20482_All	DNA polymerase alpha subunit B	8.00E-43	down
Unigene29214_All	DNA polymerase delta catalytic subunit	2.00E-55	down
Unigene30180_All	wsv514	0	up
Unigene30228_All	wsv447	0	up

### 4 Analysis of Some Interested Genes from the Transcriptomic Data

Some interesting genes, including actin binding or inhibiting protein genes, ADAM9 (disintegrin and metalloproteinase domain-containing protein 9), crustin as well as HSP70, HSP90 and GRP78 (glucose-regulated protein 78) were manually screened and the expression levels of part of the above genes were analyzed by quantitative real-time PCR. The expression patterns of these genes in Group WT at 12 hptr compared to Group W were consistent with the transcriptomic data, suggesting that the transcriptomic data is credible and can reflect the actual gene expression pattern.

Actin and integrin were regarded as possible WSSV receptors [6], [54]. In the present study, DEGs annotated as actin binding or inhibiting protein and a DEG annotated as ADAM9 were all down-regulated at the initial stage of WSSV acute infection (File S9). ADMA9 were reported to be able to bind to integrin [55], [56]. Thus, these genes might compete with WSSV for the binding to actin or disintegrin. It was guessed that the down-regulation of these DEGs was possibly resulted from temperature rising and their down-regulation was responsible for the breakout of WSSV acute infection. Therefore, the expression profiles of two actin binding genes (Unigene7524_All, and Unigene42358_All) and the ADMA9 (Unigene37466_All) in the three groups at the sampling time were detected by real-time PCR to analyze whether the down-regulation of these genes was an inducement or a result of WSSV acute proliferation.

**Figure 7:**
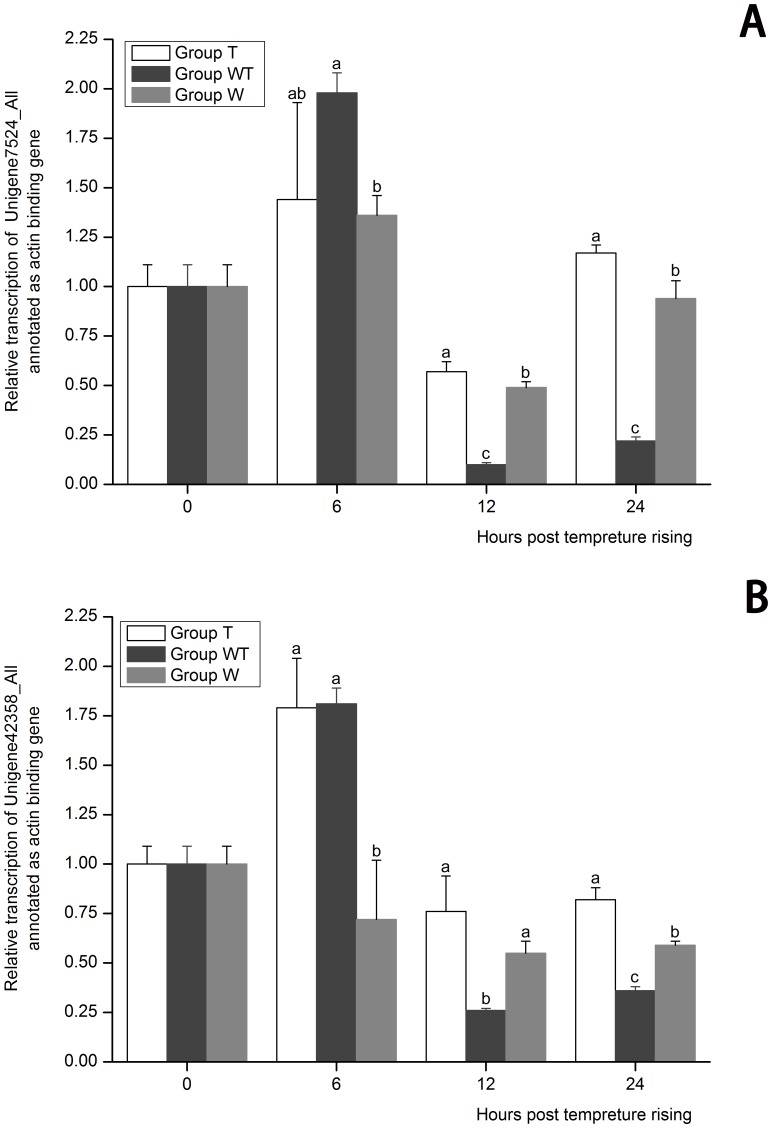
The expression profiles of Unigene7524_All (A) and Unigene42358_All (B) both annotated as actin binding gene. Their expression in Group T, Group W and Group WT at 6, 12 and 24 hours post temperature rising was tested by real-time PCR. The values are shown as mean±SE. Significant differences among the three groups are indicated by different characters (*P*<0.05).

The expression of the two actin binding genes (Unigene7524_All and Unigene42358_All) in Group TW were significantly lower than that in Group T and Group W at both 12 hptr and 24 hptr (*P*<0.05) and no significant expression differences of the two genes were observed between Group T and Group W (*P*>0.05) (Fig. 7). These results indicated that their expression had been repressed as a result of the rapid replication of WSSV.

**Figure 8:**
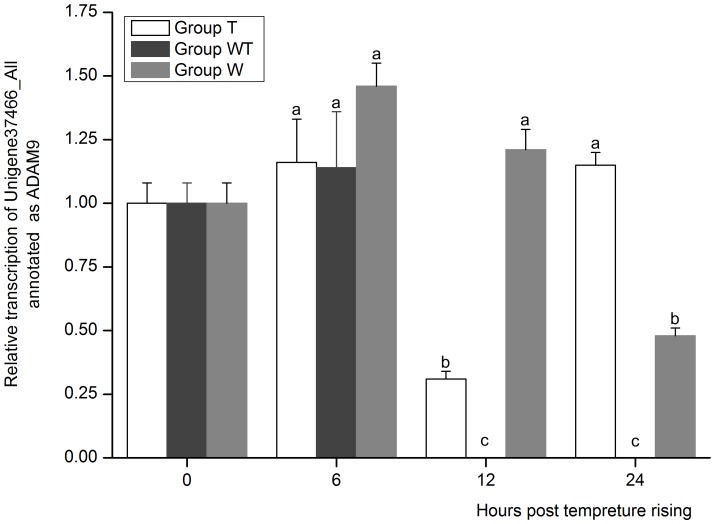
The expression profile of Unigene37466_All annotated as disintegrin and metalloproteinase domain-containing protein 9 (ADAM9). Its expression in Group T, Group W and Group WT at 6, 12 and 24 hours post temperature rising was tested by real-time PCR. The values are shown as mean±SE. Significant differences among the three groups are indicated by different characters (*P*<0.05).

The expression of ADMA9 was completely blocked in GroupWT at 12 hptr and 24 hptr and different with the expression of the above two actin binding genes, its expression in Group T at 12 hptr were also significantly lower than that in Group W (*P*<0.05) (Fig. 8), indicating that temperature rising resulted in the down-regulation of the expression of ADMA9 at 12 hptr. It was supposed that the down-regulation of ADMA9 after temperature rising resulted in the release of WSSV receptor integrin, bringing about the acute proliferation of WSSV and the expression of ADMA9 was further completely interdicted for the need of more integrin receptors in WSSV acute infection. So ADMA9 might be a possible inducer of WSSV acute infection.

**Figure 9:**
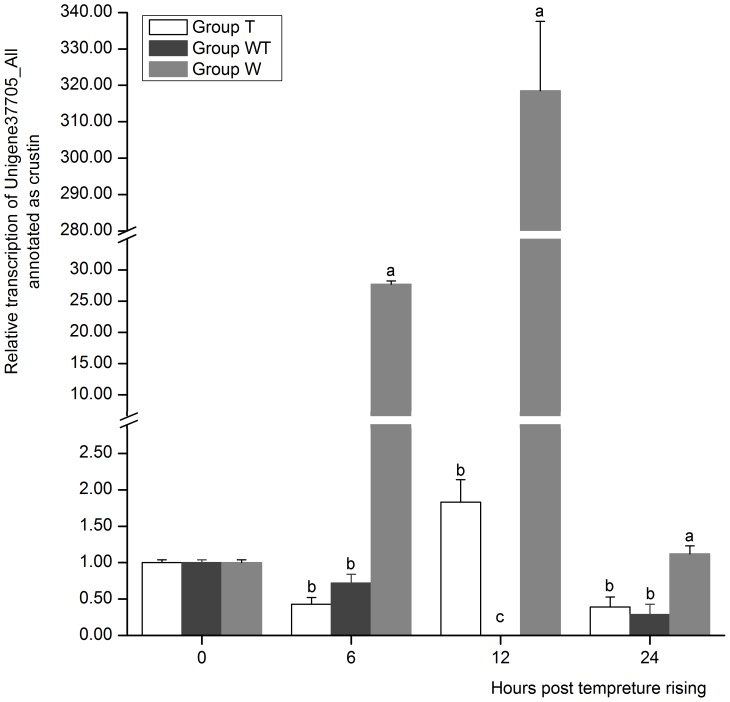
The expression profile of Unigene37705_All annotated as crustin. Its expression in Group T, Group W and Group WT at 6, 12 and 24 hours post temperature rising was tested by real-time PCR. The values are shown as mean±SE. Significant differences among the three groups are indicated by different characters (*P*<0.05).

It was interesting that 6 out of 7 crustin genes in the DEGs were down regulated and one of them (Unigene37705_All) was completely repressed (File S10). When we detected the expression of Unigenes37705 by real-time PCR, we found that its expression was completely inhibited in Group WT at 12 hptr, the initial stage of WSSV acute infection. By contrast, it was significantly up-regulated in Group W at 6 hptr and 12 hptr, when the shrimp of this group were still at the stage of latent infection (Fig. 9). Crustin is a widely distributed family of antimicrobial peptide within decapod of crustaceans and was regarded as an important component of the innate immune system [57]. Most reports about crustins focused on their anti-bacterial function and little is known about their interaction with virus. A significant down-regulation of the crustin-like AMP was noted post WSSV challenge in *Penaeus monodon*. After administrating immunostimulants, such as the marine yeast *Candida haemulonii*, the expression of crustin-like AMP was enhanced, and the shrimp showed increased viability against WSSV infection [58]. In *Macrobrachium rosenbergii*, which has much stronger resistance to WSSV than penaeid shrimp, the expression of crustin remained up-regulation during WSSV infection [59]. Together with this study, it could be indicated that crustin may function as a WSSV-resistant gene and was possibly a responsible factor for the slowed infection pace of Group W. The virulence of viruses is partially related to their ability to suppress the host anti-viral defense [29], and the results demonstrated that WSSV is highly pathogenic.

**Figure 10:**
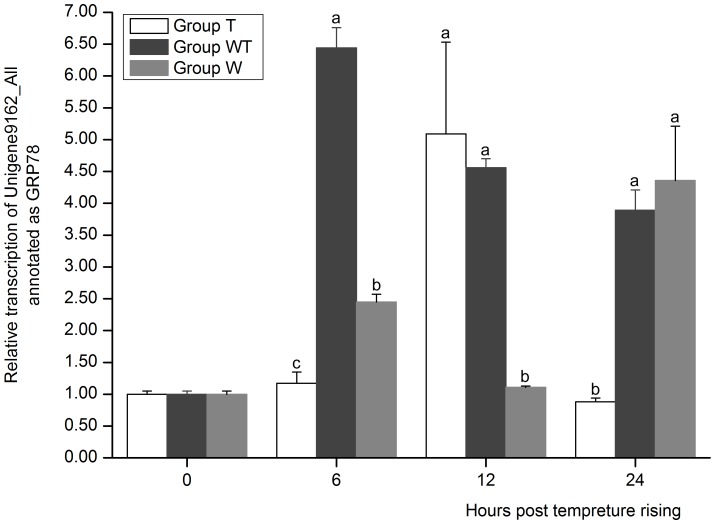
The expression profile of Unigene9162_All as annoted as GRP78. Its expression in Group T, Group W and Group WT at 6, 12 and 24 hours post temperature rising was tested by real-time PCR. The values are shown as mean±SE. Significant differences among the three groups are indicated by different characters (*P*<0.05).

It is also very interesting to note that HSP70, HSP90 and glucose-regulated protein 78 (GRP78) genes were all up-regulated in Group WT at 12 hptr (File S11). Heat-shock cognate protein 70 (Hsc70) of shrimp was a binding partner of WSSV envelop protein VP28 during infection and their association was specific, ATP-dependent and Hsc70 concentration dependent [60]. Hsp70 could inhibit WSSV replication at high temperature [20]. Thus the roles of these heat shock proteins during WSSV infection deserve much attention. The expression profile of one GRP78 gene (Unigene9162_All) was detected in the three groups at the sampling time (Fig. 10). At 6 hptr, the expression of GRP78 in GroupWT was significantly higher than that in Group T (*P*<0.05) and its high expression level in Group WT remained thereafter. This result was consistent with previous report that GRP78 kept a high expression level in hepatopancreas all through the WSSV infection course [61]. At 24 hptr, its expression in Group WT and Group W were all significantly higher than that in Group T (*P*<0.05). These results implied that the up-regulated expression of this GRP78 gene was due to WSSV infection. GRP78 functions not only in unfolded protein response and oxidative stress, but also in anti-apoptosis process [62], [63]. The up-regulation of GRP78 was induced by the infection of many other viruses including hepatitis B virus (HBV), hepatitis C virus, HCMV, rotavirus and Dengue virus (DENV) [64]–[67]. However it exhibits distinct functions in different virus infections. GRP78 acts as an endogenous antiviral factor against HBV [65], whereas it could be annexed by other viruses to facilitate the infection. GRP78 may be utilized as a chaperone for viral-protein production by DENV, for virion release by HMCV and for infectious virion maturation by rotavirus [66]. The GRP78 was activated during WSSV infection but further study is needed to clarify whether it was involved in WSSV pathogenesis or in shrimp immunity against the virus.

In summary, through the expression analyses of all the above genes as well as some other ones in Group W and Group WT at 12 hptr by real-time PCR, which were consistent with the transcriptomic data, it was testified that the transcriptomic data are creditable. At the initial stage of WSSV acute infection after temperature rising from 18°C to 25°C, WSSV exploited the cellular machinery for the seek of its own proliferation and the shrimp went through a lot of biological changes as this study demonstrated. For more information about the inducing mechanisms of WSSV acute infection after temperature change, sampling had better be carried out earlier when the infection of Group WT was at the end of the latent stage and the virus hasn’t undertaken rapid replication. Understanding the interactions between the host and WSSV at the initial stage of acute infection will not only help to get a deep insight into the pathogenesis of WSSV but also provide clues for therapies.

## Supporting Information

File S1 **File submitted to the website for GO analysis of up-regulated DEGs.**
(OUT)

File S2 **File submitted to the website for GO analysis of down-regulated DEGs.**
(OUT)

File S3 **All the DEGs used in this research.**
(XLS)

File S4 **DEGs identical to WSSV genome.**
(XLS)

File S5 **DEGs used for analysis of TCA and glycosis.**
(XLS)

File S6 **DEGs annotated as E3 ubiquin-protein.**
(XLS)

File S7 **DEGs annotated as cyclin.**
(XLS)

File S8 **DEGs used for analysis of DNA synthesis process.**
(XLS)

File S9 **DEGs annotated as actin binding or suppressor gene and a DEG annotated as ADAM gene.**
(XLS)

File S10 **DEGs annotated as crustin.**
(XLS)

File S11 **DEGs annotated as GRP78, HSP70 or HSP90.**
(XLS)
